# Integrated metabolome and immunity analysis of immune-physiological responses in dairy cows under heat stress condition

**DOI:** 10.5713/ab.25.0038

**Published:** 2025-05-12

**Authors:** Jun Sik Eom, Sangjin Lee, Joonpyo Oh, Byeong Cheol Ban, Yeeun Kim, Goeun Han, Bon-Hee Gu, Eun-Tae Kim, Sang-Bum Kim, Sung Sill Lee, Myunghoo Kim

**Affiliations:** 1 Dairy Science Division, National Institute of Animal Science, Rural Development Administration, Cheonan, Korea; 2 Institute of Agriculture and Life Science, Gyeongsang National University, Jinju, Korea; 3 Department of Animal Science, College of Natural Resources & Life Science, Pusan National University, Miryang, Korea; 4 Cargill Animal Nutrition, Seongnam, Korea; 5 Life and Industry Convergence Research Institute, Pusan National University, Miryang, Korea; 6 Subtropical Livestock Research Institute, National Institute of Animal Science, Rural Development Administration, Jeju, Korea; 7 Institute for Future Earth, JYS Institute for Basic Science, Pusan National University, Busan, Korea

**Keywords:** Heat Stress, Immune Response, Jersey Dairy Cow, Metabolomics, Peripheral Blood Mononuclear Cell

## Abstract

**Objective:**

This study aimed to investigate the metabolic changes and immune responses in Jersey dairy cows under heat stress (HS). The focus was on understanding HS-induced alterations in metabolism and immunity compared to HS-free cows.

**Methods:**

Proton nuclear magnetic resonance spectroscopy-based metabolomics was performed on rumen fluid, serum, and milk samples which were collected through single sampling from HS-free (n = 9) and HS-exposed (n = 8) Jersey cows. Immune cell proportions and functions in peripheral blood mononuclear cells (PBMC) were analyzed to identify heat-sensitive immunological changes. Correlation analysis was conducted to link immune indicators with serum metabolites.

**Results:**

Metabolomics revealed potential HS biomarkers in biofluids: nicotinate and phenylacetate in rumen fluid; isopropanol in serum; and glycine and trehalose in milk. HS increased B cell and CD4+ T cell populations, as well as Th17 cells and IL-17A expression in PBMCs. Twenty-three metabolites correlated strongly with five immune indicators, with nine metabolites exhibiting a positive correlation and fourteen a negative correlation.

**Conclusion:**

HS alters the both metabolism and immune cell function in Jersey dairy cows. These findings provide key information for the development of diagnostic methods for HS and nutritional intervention strategies to mitigate HS for dairy cows.

## INTRODUCTION

The dairy farming industry is highly vulnerable to the effects of global warming and climate change. Specifically, dairy cows are more vulnerable to heat stress (HS) than other farm animals. Previous studies have reported various negative physiological responses to HS in dairy cows when the average temperature-humidity index (THI) exceeds 68 (equivalent to ~22°C at 50% relative humidity) [1], such as growth performance, milk quality, and production [2–4]. In addition, HS can adversely affect the metabolism, physiology, and immune system of dairy cows. Skibiel et al [5] reported that dairy cows under HS conditions show various abnormal responses, including mitochondrial dysfunction, increased oxidative stress, hepatic lipid accumulation, and shifts in the precursor supply for gluconeogenesis in the liver. Moreover, HS exposed dairy cows showed impairments in lipid metabolism in bovine primary adipocytes, carbohydrate and lipid metabolism, milk protein synthesis in mammary tissues, glucose and energy metabolism, energy balance, and high susceptibility to infectious diseases like mastitis and metritis [6–8]. Recently, it has been suggested that altered metabolism or metabolites are the major causes of immune-related diseases. For example, various metabolites such as short chain fatty acids and bile acids regulate immune cell function and differentiation during disease development [9,10]. Therefore, increasing our understanding of the effect of HS on biological fluid metabolism and immune responses in dairy cows is a major area of interest.

The emergence of omics techniques has created a new path for studying intricate biological processes, along with the potential to increase our understanding by facilitating the measurement of a multitude of molecules. Metabolomics research aims to understand changes in physiological and biochemical status, with metabolic profile identification and quantification of various biological fluids. Metabolomics recently used to understand altered metabolism and physiology in cows with HS [11–13]. Dairy cows are at risk of exposure to metabolic diseases (e.g., ketosis, metritis) before and after calving, which can lead to decreased milk production, reduced reproductive ability, premature culling, and economic losses in the dairy farming industry. Metabolomics research on early diagnosis and prediction of metabolic diseases in dairy cows is actively underway. Identifying metabolic biomarkers in biological fluids associated with metabolic diseases helps develop a predictive diagnosis and early intervention for improving dairy cow health and welfare. Recent studies have reported that HS affects physiological parameters and alters the metabolic composition of biological fluids in dairy cows [14].

While numerous studies have investigated metabolic changes induced by HS in Holstein cows, research on Jersey cows remains limited. Also, limited range of assessment for metabolites and immunity has been performed in heat stressed dairy cows. This study provides much knowledge for metabolic and immunity under HS conditions, offering new insights into the systemic immune-metabolic interplay in Jersey cows. We hypothesized that HS induces metabolic and immune changes in Jersy cows, and there are potential links between altered metabolites and immune parameters. The objective of this study was to investigate the changes of metabolites and immune parameters in Jersey cows under HS environmental condition. To understand changes in the biological responses of dairy cows to HS, this study examined the metabolite profiles of three biological fluids (rumen fluid, serum, and milk) and immunological parameters of peripheral blood mononuclear cells (PBMCs) in Jersey cows, with a focus on metabolism, immune responses, and their potential interactions.

Proton nuclear magnetic resonance (^1^H-NMR) spectroscopy, and flow cytometry and quantitative real-time reverse-transcription polymerase chain reaction (qRT-PCR) were conducted under an optimum temperature period (OTP) and high temperature period (HTP), respectively. The results of our integrative analysis will help develop new strategies for discovering novel diagnostic biomarkers for diseases and dietary interventions for ameliorating the adverse effects of HS on Jersey cows.

## MATERIALS AND METHODS

### Experimental design, animals, and diet

Seventeen Jersey cows from the Seoul milk biotechnology laboratory were used in study (OTP, n = 9, 44.1±10.8 months old; HTP, n = 8, 33.9±5.8 months old). All cows were kept in stalls with feeding and water facilities and fed basal total mixed ration (TMR) was once a day at 08:00. The experimental diet consisted of TMR provided to meet nutrient requirements according to National Research Council (NRC) recommendations [15].

The rumen fluid, blood, and milk samples were collected under different THI. With respect to livestock, THI is mainly used to reflect the intensity of HS. Accordingly, based on the available meteorological data, we used THI to examine the effects of HS on Jersey cows. To measure the degree of HS in Jersey cows, two time slots according to the THI were assessed for comparative studies. For the determination of THI, we measured temperature and humidity using a temperature and humidity meter (Testo 174H Mini data logger; Testo, West Chester, PA, USA) and applied the following THI equation, devised by NRC (1971) [15].

“THI = (1.8 × ambient temperature + 32) − [(0.55 − 0.0055 × relative humidity) × (1.8 × ambient temperature − 26)]” Jersey cows were not exposed to direct sunlight during HTP, and no additional measures were implemented beyond standard experimental protocols. If a cow shows severe HS symptoms, veterinary treatment was applied, and the animal was excluded from the study. There was no exclusion in the study.

### Feed sampling and analysis

Dried feed samples (TMR and concentrate) were ground through a 1 mm sieve using a Wiley Mill (Arthur Thomas, Philadelphia, PA, USA) and submitted to Cumberland Valley Analytical Services (Waynesboro, PA, USA) to be analyzed by wet chemistry methods for dry matter (AOAC International method 930.15), crude protein (CP, AOAC International method 990.03), ether extract (EE, AOAC International method 2003.05), ash (AOAC International, 2000; method 942.05), minerals (AOAC International method 985.01), amylase-treated neutral detergent fiber (aNDF) [16], acid detergent fiber (AOAC International method 973.18), neutral detergent insoluble crude protein (NDICP, Leco FP-528 N Combustion Analyzer), acid detergent insoluble crude protein (Leco FP-528 N Combustion Analyzer), lignin, and starch. Non-fiber carbohydrates (NFC) were calculated according to the equation by Hall [17]; NFC = 100–[(CP–NDICP)+EE+ash+NDF]. Net energy for maintenance was calculated using the OARDC Summative Energy Equation, as described by Weiss [18]. The chemical composition of the TMR and commercial concentrate are presented in Table 1.

### Rumen fluid, blood and milk sampling analyses

Rumen fluid, blood, and milk samples were collected before morning feeding. Rumen fluid was collected from Jersey cows using a stomach tube (OTP, n = 9; HTP, n = 8). Samples were collected once from each Jersey cow, and the selected time point was considered representative of metabolic and immune responses under HS. The first rumen fluid was not sampled because of saliva and blood contamination. The samples were centrifuged at 806×g at 4°C for 15 min to remove feed particles, and the supernatant was stored at −80°C for ^1^H-NMR spectroscopy analysis. Blood from the jugular neck vein was collected in a serum-separating tube (SST) (BD Vacutainer, SST II advance; Becton Dickinson, Franklin Lakes, NJ, USA) from Jersey cows (OTP, n = 9; HTP, n = 8). The blood samples were centrifuged at 806×g at 4°C for 15 min, and aliquots of the upper layer (serum) were stored at −80°C for ^1^H-NMR spectroscopy analysis. Finally, milk samples were collected by using pipeline milking system and then transferred to conical tubes (30 mL each) (OTP, n = 9; HTP, n = 8). Samples were stored at −80°C for ^1^H-NMR spectroscopy analysis.

### Sample preparation and analysis for ^1^H-NMR spectroscopy

The rumen fluid samples were centrifuged at 12,902×g at 4°C for 10 min, and 300 μL of the supernatant was collected. A standard buffer solution (2,2,3,3-d(4)-3-(trimethylsilyl) propionic acid [TSP] sodium salt) was added to 300 μL of the supernatant in deuterium oxide (D_2_O) solvent/standard buffer solution (300 μL). The supernatants (600 μL) were transferred to 5 mm NMR tubes for ^1^H-NMR spectroscopy analysis. We prepared saline buffer (concentration of 0.9% wt/vol) by applying NaCl in 100% D_2_O solvent. The stored serum samples were centrifuged at 14,000×g at 4°C for 10 min. The supernatant (200 μL) was added to 400 μL of saline buffer in 5 mm NMR tube for ^1^H-NMR spectroscopy analysis. Milk samples were centrifuged at 4,000 × g at 4°C for 15 min to remove the lipid layer in supernatant. Thereafter, the mixture of milk (250 μL) and D_2_O solvent (300 μL) were transferred to 5 mm NMR tube for ^1^H-NMR spectroscopy analysis.

The spectra of rumen fluid, serum and milk samples were obtained on a SPE-800 MHz NMR-MS Spectrometer (Bruker BioSpin AG, Fällanden, Switzerland) equipped with a 5 mm triple-resonance inverse cryoprobe with Z-gradients (Bruker BioSpin, Billerica, MA, USA). The pulse sequence used for the two samples (rumen fluid and serum) and milk were a NOESY presaturation and Carr-Purcell-Meiboom-Gill pulse sequence, respectively. We collected 64,000 data points with 128 transients, a spectral width of 16,025.641 Hz, a relaxation delay of 4.0 s, and an acquisition time of 2.0 s.

### Metabolites identification, quantification and statistical analyses

Metabolite identification and quantification were carried out by importing the analyzed spectral data into the Chenomx NMR suite 8.4 software (ChenomxInc, Edmonton, AB, Canada). The baseline and phase were matched for comparison between samples using the Chenomx processor. The spectral width was δ 0.2 to 10.0 mg/kg and was referenced to the TSP signal at 0.0 mg/kg. Qualitative and quantitative metabolite analyses were performed using the Livestock Metabolome Database ( http://www.lmdb.ca), Bovine Metabolome Database ( http://www.bmdb.ca), and the Chenomx profiler. Statistical analyses of the metabolite data were performed using MetaboAnalyst 5.0 ( http://www.metaboanalyst.ca). To perform a standard cross sectional two periods study, we compared the periods of OTP and HTP conditions. The resulting data were processed by normalization-selected methods, followed by sample normalization via normalization to constant median, data transformation via log normalization, and data scaling via auto scaling. The rumen fluid, serum, and milk metabolite data with 50% of samples under the identification limit or with at least 50% of values missing were eliminated from the analysis. The metabolites that were missing positive in the original data. The univariate Student’s t-test was used to quantify differences between in the metabolite profiles of the rumen fluid, serum, and milk under the OTP and HTP conditions.

Principal components analysis (PCA) and partial least square-discriminant analysis (PLS-DA) were used as multivariate data analysis techniques to generate a classification model and provide quantitative information for discriminating rumen fluid and serum metabolites. The different periods of rumen fluid, serum, and milk metabolites from OTP and HTP conditions were determined based on a statistically significant threshold of variable importance in projection (VIP) scores. Metabolites with VIP scores higher than 1.5 were obtained using the PLS-DA model. Metabolic pathway analysis was performed using the *Bos taurus* pathway library (Kyoto Encyclopedia of Genes and Genomes [KEGG], http://www.kegg.com). Significantly different metabolic pathways in the rumen fluid, serum, and milk metabolites of the other study animals were statistically analyzed and determined using MetaboAnalyst 5.0, which is based on the database source by KEGG.

### Peripheral blood mononuclear cells isolation

Blood samples were collected from the jugular vein of Jersey and contained using K2-EDTA tubes and SST tubes (BD Vacutainer; Becton Dickinson). EDTA tube was used for PBMC separation and SST tube was used for serum separation. About 20 mL of blood was collected per animal. The collected blood was immediately transferred to the ice and immediately moved to the laboratory. To separate the serum, the whole blood in the SST tube were subjected to centrifugation at 3,000×g for 15 min at room temperature and harvested upper supernatant. All serum samples stored at −80°C until biochemistry analysis. The whole blood from EDTA tubes was utilized for PBMC isolation. Density gradient centrifugation was used to isolate the PBMCs. Briefly, whole blood samples were diluted with PBS in a 1:1 ratio in 15 mL conical tubes. Then, diluted blood samples were applied to a lymphoprep (STEMCELL Technologies, Vancouver, BC, Canada) gradient (8 mL of diluted blood on the top and 4 mL of lymphoprep on the bottom) by centrifugation for 30 min at 800×g without breaking at room temperature. After centrifugation, The PBMCs from the middle layer was collected and washed with PBS to obtain purified PBMC.

### Flow cytometry analysis of Jersey dairy cow peripheral blood mononuclear cells

The PBMCs isolated from Jersey cows during OTP and HTP were subjected to immune cell population quantification using flow cytometry (FACS Canto II; BD Biosciences, Heidelberg, Germany). Briefly, the PBMC fixed using 4% paraformaldehyde for 20 min at 4°C and stained with direct fluorescence conjugated antibodies; anti-CD4:Alexa Flour 647 (Bio-Rad, MCA1653A647), anti-CD21:PE (Bio-Rad, MCA1424PE), anti-CD172a:PE-Cy5 (Bio-Rad, MCA2041C), anti-WC1:FITC (Bio-Rad, MCA838F), anti-MHCII:FITC (Bio-Rad, MCA5656F), anti-CD16:FITC (Bio-Rad, MCA5665F), anti-CD14:PE (Bio-Rad, MCA1568PE), anti-CD8:PE (Bio-Rad, MCA837PE), anti-IFNg:FITC (Bio-Rad, MCA1783A488), anti-IL-17A:Biotin (Bio-Rad, AHP2376B), and Streptavidin: PEcy5 (Invitrogen, 45-4317-80). All antibodies were diluted to 1:200 in PBS. The analysis was conducted by 3 panel groups: 1) CD172a, CD14, and CD26 antibodies for monocytes, and 2) CD21, MHCII, CD4, CD8, and WC1 antibodies for CD4 T cells, CD8 T cells, γδ T cells, and B cells; 3) CD4, IFNg, IL-17A, and Streptavidin antibodies for Th1 and Th17 cells.

### Real-time quantitative polymerase chain reaction

Total RNA was isolated from cell samples to examine the respective responses in Jersey dairy cows. Samples in Trizol were incubated for 5 min at room temperature, after which 200 μL of chloroform was added to 1 mL of Trizol. The samples were then vortexed for 10 s and incubated for a further 2 min at room temperature for phase separation. Subsequently, the samples were centrifuged at 10,000×g for 20 min at 4°C. The resulting upper aqueous phase was transferred into fresh tubes, to which 0.5 to 1 mL of isopropyl alcohol was added, followed by gentle mixing by shaking. After incubation for 10 min at room temperature and centrifugation at 10,000×g for 10 min at 4°C, the supernatant was removed, and the resulting RNA pellet was washed with 75% ethanol prior to being stored in Diethyl pyrocarbonate (DEPC) water (Invitrogen, Carlsbad, CA, USA). cDNA was synthesized using AccuPower RT PreMix (Bioneer, Daejeon, Korea). qRT-PCR was performed using a QuantStudio1 Real-Time PCR system (Applied Biosystems, Foster City, CA, USA) and SologTM h-Taq DNA Polymerase (SolGent, Daejeon, Korea). The reaction conditions were as follows: 50°C for 10 min, 95°C for 5 min, 95°C for 15s, and 60°C for 30 s (40 cycles), followed by melting curve analysis. The expression levels of gene were normalized to the β-ACTIN mRNA level. Confirmed sequences of primer pairs for quantitative RT-PCR were as follows: forward 5′-AGC AAG CAG GAG TAC GAT GAG T-3′ and Reverse 5′-ATC CAA CCG ACT GCT GTC A-3′ for β-ACTIN; forward 5′-TGA GTC TGG TGG CTC TTG TG-3′ and Reverse 5′-GGT GGA GCG CTT GTG ATA AT-3′ for IL-17A; forward 5′-GAT TCA AAT TCC GGT GGA TG-3′ and Reverse 5′-AAA TAT TGC AGG CAG GAG GA-3′ for IFN-γ.

### Correlation analysis

Correlation analysis was conducted to examine the associations between serum metabolites and immune indicators. Metabolite data were obtained from the serum of Jersey cows (n = 17) during the OTP and HTP periods. To enhance statistical reliability, only metabolites detected in at least five serum samples per group were included in the analysis. Immune indicator data corresponding to these selected metabolites were incorporated into the analysis. Spearman’s correlation analysis was subsequently performed to evaluate the strength and direction of these associations. Hash on the graph represents level of significance (p<0.05).

## RESULTS

### Identified and quantified metabolite classification in three biological fluid of Jersey cows

^1^H-NMR spectroscopy in three biological fluids (rumen fluid, serum, and milk) from Jersey cows are summarized in Supplements 1–3. In rumen fluid, 158 metabolites were detected and 73 quantified under OTP, while 155 were detected and 78 quantified under HTP. Serum samples revealed 122 detected and 52 quantified metabolites in OTP, compared to 104 detected and 49 quantified in HTP. Milk samples had 123 detected and 56 quantified metabolites in OTP, and 107 detected and 33 quantified in HTP. Common metabolites among the biological fluids under OTP and HTP are illustrated in Supplement 3. For OTP, 66 metabolites were shared across all three biological fluids, while for HTP, 59 metabolites were shared. Regarding quantified metabolites, 17 were common across all biofluids in OTP, and 13 in HTP.

### Multivariate statistical analysis of biological fluid metabolites in Jersey cows

**Figure 1:**
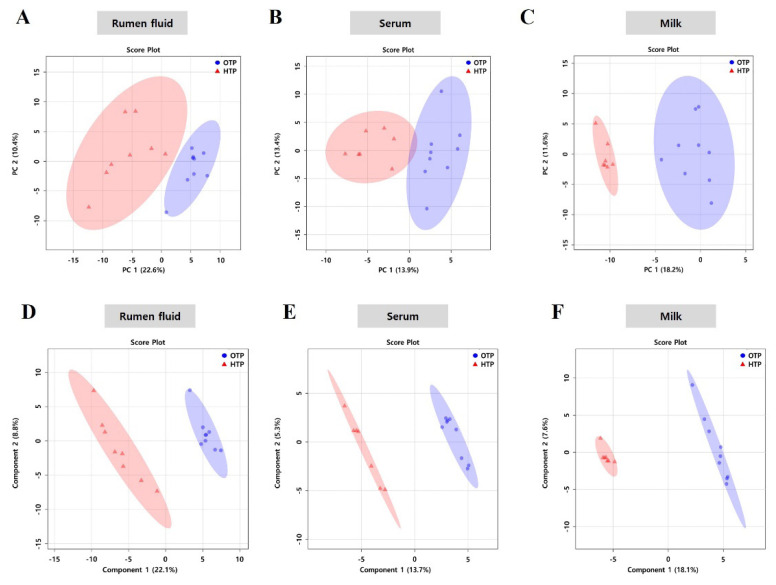
Principal components analysis (PCA) and partial least square-discriminant analysis (PLS-DA) score plot of metabolites in biological fluids of Jersey cows under optimum temperature period (OTP) and high temperature period (HTP) conditions. PCA was performed on rumen fluid (A), serum (B), and milk (C) samples and PLS-DA was performed on rumen fluid (D), serum (E), and milk (F) samples from Jersey cows under OTP and HTP conditions. On the score plot, each point represents individual samples, with blue dot representing OTP (n = 9) and red triangle representing the HTP (n = 8). The x-axis and y-axis represent the variance associated with PC 1 and 2 in PCA and component 1 and 2 in PLS-DA, respectively.

Based on the data obtained from ^1^H-NMR spectroscopy, PCA and PLS-DA (Figure 1) of rumen fluid, serum, and milk samples were used to interpret and obtain metabolic profiles. We observed a highly clustered sample collected during OTP, which was distinct from that observed in the biological fluids collected during HTP. The PCA score plots for rumen fluid (PC 1: 22.6% and PC 2: 10.4%) and milk (PC 1: 18.2% and PC 2: 11.6%) showed dramatic changes in metabolic profiles (Figures 1A, 1C). In contrast, metabolites from the serum (PC 1: 13.9% and PC 2: 13.4%) did not show a clear distinction (Figure 1B). In the PLS-DA score plots, the samples were clearly demarcated into two parts as the THI changed from OTP to HTP, which indicated changes in the metabolic profiles of the three biological fluid samples (rumen fluid, component 1: 22.1% and component 2:8.8%; serum, component 1: 13.7% and component 2: 5.3%; milk, component 1: 18.1% and component 2: 7.6%). In addition, we observed less variation in the metabolites from the rumen fluid and serum samples obtained during OTP compared to those observed in samples obtained during HTP (Figures 1D–1F).

### Differentially expressed metabolites in three biological fluids of Jersey cow in optimum temperature period and high temperature period

**Figure 2:**
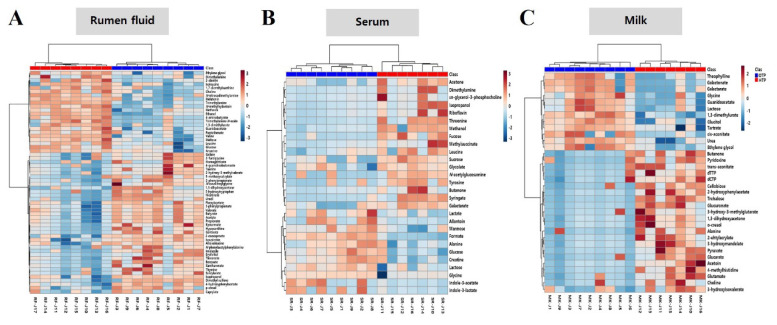
Heatmap analysis for differential metabolites in biological fluids of Jersey cows under optimum temperature period (n = 9) and high temperature period (n = 8) conditions. Heatmap analysis of differential metabolites in rumen fluid (A), serum (B), and milk (C) obtained by Student’s t-test model analysis (p<0.05). The red and blue colors in the plot describe high and low intensities, and the value ranges from −3 to +3.

Differentially expressed metabolites in samples of the three biological fluids were investigated based on their relative intensities during OTP and HTP. The metabolomic profiles of the three biological fluid generally clustered together under OTP or HTP (Figure 2; Supplementas 4–6). In the rumen fluid samples, nicotinate, uracil, imidazole, acetate, and hypoxanthine levels were significantly higher in OTP than in HTP (p<0.05). In contrast, trimethylamine, ethanol, *N*-methylhydantoin, 4-aminobutyrate, and valine levels were significantly higher in HTP than in OTP (p<0.05) (Figure 2A; Supplement 4). In the serum samples, formate, glucose, creatine, lactose, and mannose levels were significantly higher in OTP than in HTP (p<0.05). Conversely, threonine, fucose, syringate, acetone, and sucrose levels were significantly higher in HTP than in OTP (p<0.05) (Figure 2B; Supplement 5). In milk samples, 1,3-dimethylurate, galactonate, glucitol, urea, and guanidoacetate levels were significantly higher in OTP than in HTP (p<0.05), whereas trehalose, glucuronate, 4-methylhistidine, 2-hydroxyphenylacetate, and pyruvate levels were significantly higher in HTP than in OTP (p<0.05) (Figure 2C; Supplement 6).

**Figure 3:**
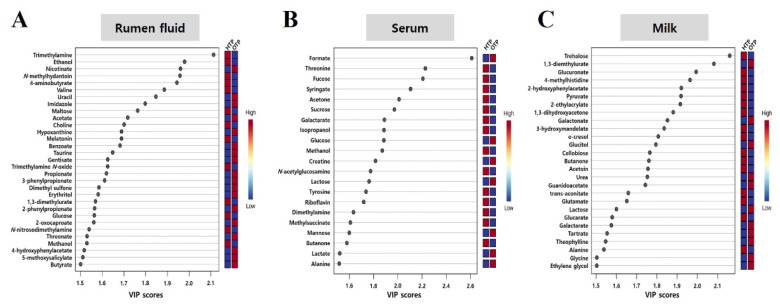
Variable importance in projection (VIP) scores for differential metabolites in biological fluids of Jersey cows between optimum temperature period (OTP) and high temperature period conditions (HTP). VIP score for differential metabolites in rumen fluid (A), serum (B), and milk (C) of Jersey cows under OTP (n = 9) and HTP (n = 8) conditions. The selected metabolites were those with VIP score>1.5. Heat map with red or blue boxes on the right indicates high and low abundance ratio, respectively, of the corresponding rumen fluid, serum, and milk metabolites in OTP and HTP conditions. The VIP score was based on the partial least square-discriminant analysis model.

According to the PLS-DA model, each metabolite in the rumen fluid, serum, and milk samples had a VIP score>1.5 (Figure 3; Supplements 4–6). In rumen fluid samples, nicotinate (VIP score: 1.96), uracil (1.85), imidazole (1.80), acetate (1.72), and hypoxanthine (1.69) had higher VIP scores in the OTP group than those in the HTP group. In contrast, trimethylamine (2.11), ethanol (1.98), *N*-methylhydantoin (1.96), 4-aminobutyrate (1.94), and valine (1.89) had higher VIP scores during HTP than those during OTP. When comparing VIP scores in the serum samples of OTP and HTP groups, formate (2.61), glucose (1.89), creatine (1.82), lactose (1.76), and mannose (1.60) had the highest VIP scores in the OTP group. In contrast, threonine (2.23), fucose (2.21), syringate (2.11), acetone (2.01), and sucrose (1.97) had higher VIP scores during HTP than during OTP. Among the milk samples, 1,3-dimethylurate (2.08), galactonate (1.85), glucitol (1.80), urea (1.75), and guanidoacetate (1.74) had higher VIP scores in the OTP group than those in the HTP group. In contrast, trehalose (2.16), glucuronate (1.99), 4-methylhistidine (1.96), 2-hydroxyphenylacetate (1.92), and pyruvate (1.92) had higher VIP scores during HTP than that of those during OTP. Our *t*-test results showed that the metabolites with the highest VIP scores were similar for all biofluids.

### Metabolic pathway analysis for comparison of biological fluid metabolomes in Jersey cows under optimum temperature period and high temperature period

**Figure 4:**
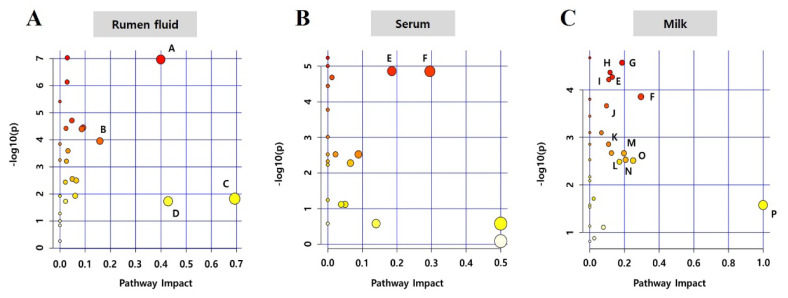
Metabolic pathway mapping significantly differs in biological fluids of Jersey cows between optimum temperature period (OTP) and high temperature period (HTP) conditions. Metabolic pathway mapping significantly differs in rumen fluid (A), serum (B), and milk (C) of Jersey cows under OTP (n = 9) and HTP (n = 8) conditions. The x-axis and y-axis represent pathway impact and -log 10 p value, respectively. The results are presented graphically as bubble plot. The darker color and larger size represent p value from enrichment analysis and greater impact from pathway topology analysis, respectively. A, beta-alanine metabolism; B, tryptophan metabolism; C, caffeine metabolism; D, taurine and hypotaurine metabolism; E. glyoxylate and dicarboxylate metabolism; F, glycine, serine and threonine metabolism; G, glycolysis /gluconeogenesis; H, arginine biosynthesis; I, arginine and proline metabolism; J, citrate cycle (TCA cycle); K, glutathione metabolism; L, pentose and glucuronate interconversions; M, alanine, aspartate and glutamate metabolism; N, pyruvate metabolism; O, ascorbate and aldarate metabolism; P, D-glutamine and D-glutamate metabolism.

The metabolome view map was analyzed to characterize significantly different metabolic pathways observed in the three biological fluids under varying THI conditions (Figure 4; Supplements 7–9). In rumen fluid, 20 metabolic pathways, in serum, 12, and in milk, 14 showed significant alterations. The following four pathways had an impact value>0.1, which is the cutoff value for relevance in rumen fluid: caffeine metabolism (impact value: 0.69), taurine and hypotaurine metabolism (0.43), beta-alanine metabolism (0.40), and tryptophan metabolism (0.16) (Figure 4A; Supplement 7). In serum, the following two pathways were observed with impact values>0.1: glycine, serine, and threonine metabolism (0.30) and glyoxylate and dicarboxylate metabolism (0.19) (Figure 4B; Supplement 8). In addition, 12 pathways were observed in milk, including D-glutamine and D-glutamate metabolism (1.00); glycine, serine, and threonine metabolism (0.30); ascorbate and aldarate metabolism (0.25); and pyruvate metabolism (0.21) (Figure 4C; Supplement 9).

Enrichment and impact pathway analyses identified several common metabolic pathways in numerous combinations of the three biological fluids. For example, glycolysis/gluconeogenesis; arginine and proline metabolism; glycine, serine, and threonine metabolism; pyruvate metabolism; and glyoxylate and dicarboxylate metabolism were identified as common pathways between all three biological fluids.

### Complete blood count of Jersey cows during optimum temperature period and high temperature period

Complete blood count analysis of Jersey dairy cows was conducted in environments in OTP and HTP. The whole blood cell count did not differ significantly between OTP and HTP (6×10^9^/l), in addition to the lymphocyte, monocyte, eosinophil, and basophil counts. However, neutrophil cell count was significantly higher during HTP than that during OTP (Supplement 10).

### Monocyte subsets in peripheral blood mononuclear cells from Jersey cows during optimum temperature period and high temperature period

**Figure 5:**
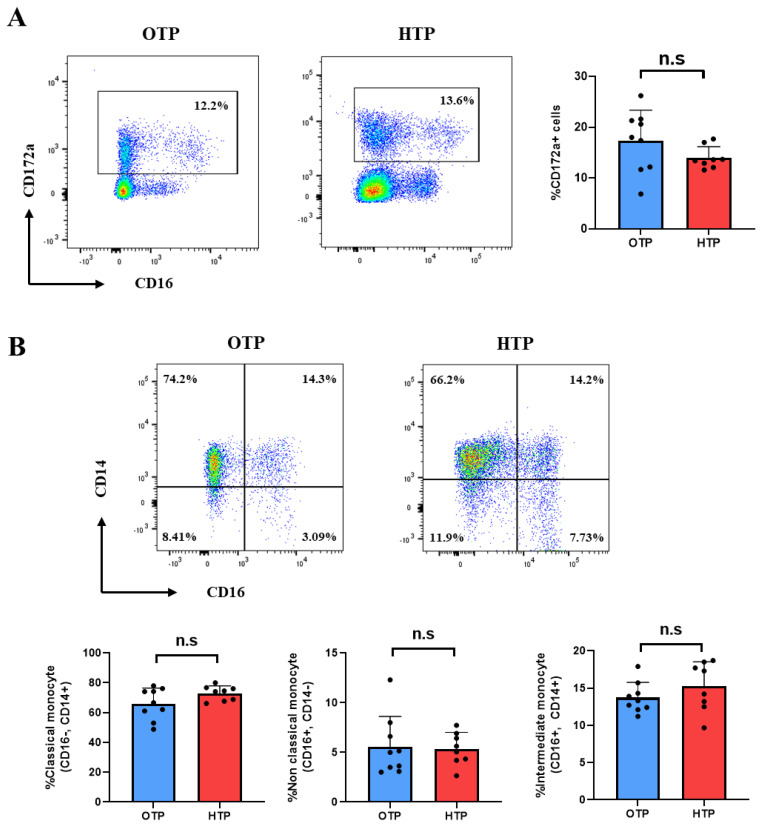
Monocyte subsets of peripheral blood mononuclear cells (PBMCs) in Jersey cows under optimum temperature period (OTP) and high temperature period (HTP) conditions. (A) Monocytes ex-pressing CD172a in PBMC assessed by flow cytometry. (B) CD172a+ monocyte is classified into three subsets by CD14 and CD16; classical CD14+CD16-, non-classical CD14-CD16+, and intermediate CD14+CD16+, monocytes. Data represented are as mean±SD; n = 8−9 animals/group. Values were statistically analyzed by Welch’s t-test.

CD172a (signal regulatory protein-α) levels in Jersey cows did not differ significantly between OTP and HTP (Figure 5A). The cow monocyte subset is divided into three categories (classical, intermediate, and non-classical monocytes), and CD14 and CD16 markers [19,20] are used to distinguish the subsets. HS had no discernible effect on classical (CD14+ CD16−), intermediate (CD14+CD16+), and non-classical monocytes (CD14-CD16+) (Figure 5B).

### Changes of T cell subsets and B lymphocytes in peripheral blood mononuclear cells from Jersey cows during high temperature period

**Figure 6:**
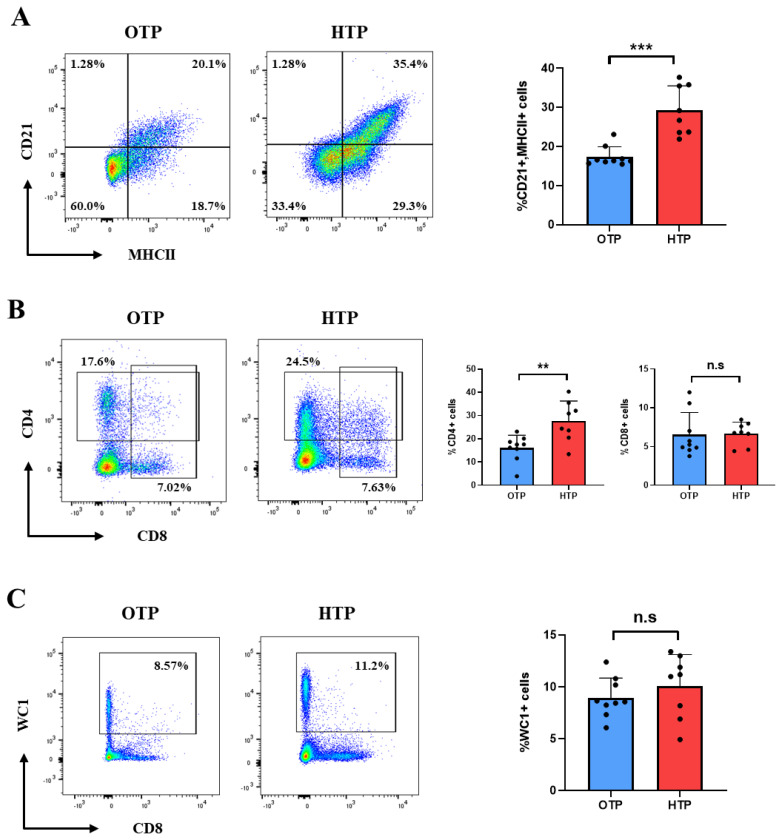
Altered B cells and T cells in peripheral blood mononuclear cells (PBMCs) from Jersey cow in optimum temperature period (OTP) and high temperature period (HTP) conditions. The populations of B cells and T cells were an-alyzed using flow cytometry. (A) B cells (MHCII+CD21+) populations, (B) CD4+ T cell (CD4+) and CD8+ T cell (CD8+) populations and (C) γδ T cell (WC1+) populations in PBMCs are shown. Data are represented as means±standard deviation (SD); n = 8–9 animals/group. Values were statistically analyzed by Welch’s t-test. * p<0.05.

The composition of T cell subsets and B cells in PBMCs from Jersey cows under different environmental conditions was examined. In PBMCs, B cells were identified as CD21+MHC II+cells. The B cell population (CD21+MHCII+) was significantly higher under HTP than that under OTP (p<0.001) (Figure 6A). T cell subsets, CD4+ T cells, CD8+ T cells, and γδ T cells in PBMCs were examined. The proportion of CD4+ T cells was significantly higher (p<0.05) in Jersey cows exposed to HTP than in those exposed to OTP (Figure 6B). However, there was no significant difference in the cell population of γδ T cells (WC1+) and CD8 T cells (CD8+) in PBMC between HTP and OTP (Figures 6B, 6C).

**Figure 7:**
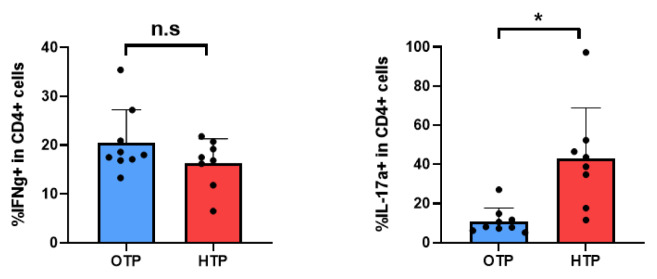
Proportion of Th1 and Th17 cells in peripheral blood mononuclear cells (PBMCs) of Jersey cows in different environmental conditions. CD4+ T cell subsets (Th1 and Th17 cells) in PBMCs from Jersey dairy cows in optimum temperature period (OTP) and high temperature period (HTP) conditions were examined by flow cytometry. Among CD4+ T cells, Th1 and Th17 cells were identified as their IFN-γ and IL-17a expression. Data are represented as means± standard deviation; n = 8–9 animals/group. Values were statistically analyzed by Welch’s t-test. * p<0.05.

In addition, CD4+ T cell subsets (Th1 and Th17) were examined. Among CD4+ T cells, IFN-γ and IL-17A-expressing T cells were identified. As shown in Figure 7, the frequency of IFN-γ expressing CD4+ T cells (Th1 cells) did not change during HTP. However, the frequency of IL-17A expressing CD4+ T cells (Th17 cells) was significantly increased in HTP in Jersey cows, in contrast to OTP (p<0.05; Figure 7). Collectively, HTP increased MHCII+CD21+ B cells, total CD4+ T cells, and Th17 cells in the blood of Jersey dairy cows.

### Changes of mRNA expression of gene for cytokine in peripheral blood mononuclear cells by high temperature period

**Figure 8:**
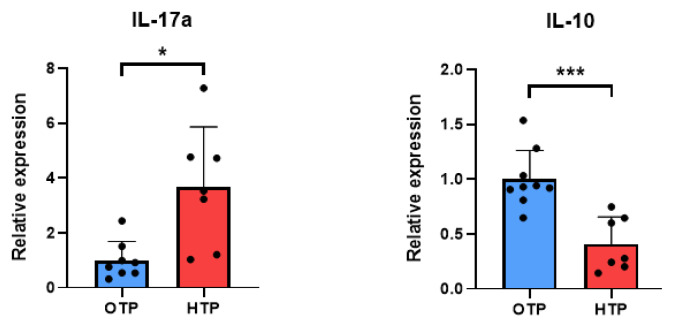
Changes of the mRNA expression of cytokines in the peripheral blood mononuclear cells (PBMCs) of Jersey dairy cows by optimum temperature period (OTP) and high temperature period (HTP) conditions. The expression of IL-17a, and IL-10 analyzed in PBMCs using qRT-PCR. Data are represented as means±standard deviation (SD); n = 8–9 animals/group. Values were statistically analyzed by Welch’s t-test. * p<0.05, *** p<0.001. qRT-PCR, quantitative reverse transcription polymerase chain reaction.

Changes in cytokine gene expression in the PBMCs of Jersey cows under HTP were examined. IL-17A is a proinflammatory cytokine and IL-10 is a representative anti-inflammatory cytokine. IL-17A gene expression significantly increased under HTP (p<0.05), whereas IL-10 expression significantly (p<0.05) decreased under the same conditions (Figure 8). Collectively, inflammatory cytokine expression increased in the PBMCs of Jersey dairy cows under HTP.

### Correlation analysis of heat stress-sensitive metabolites and immune indicators of serum in Jersey dairy cows

**Figure 9:**
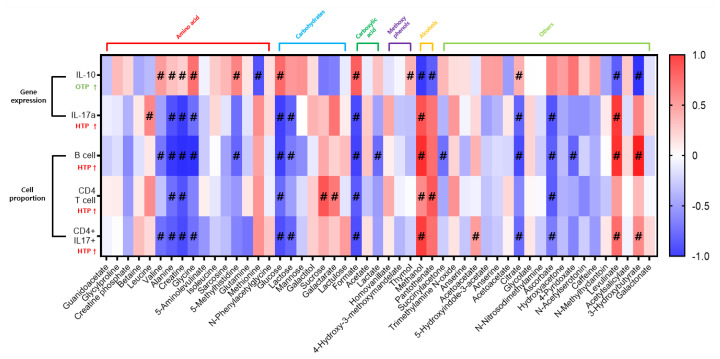
Correlation between heat response-related serum metabolites and immune indicators. Heatmap of Spearman’s correlation between serum metabolites detected in both the optimum temperature period and high temperature period condition (at least 5 in each group) and immune indicators. Red color denotes positive associations. Blue color stands for negative association. Hash on the graph represents level of significance (p<0.05). OTP, optimum temperature period; HTP, high temperature period.

Correlation analysis was performed using serum metabolites and immune indicators. To select serum metabolites, metabolite data from the serum of Jersey cows (n = 17) during the OTP and HTP periods were used. Metabolites detected in at least five serum samples per group were selected to ensure statistical significance in correlation analysis. A total of 51 metabolites were selected for analyses. Immune indicator data corresponding to the selected serum metabolites were used, including IL-10, IL-17A, B cells, CD4+ T cells, and Th17 cells, which showed significant differences between OTP and HTP. Spearman’s correlation analysis was conducted. The metabolites that were significantly correlated with immune indicators in the blood included seven amino acid (AA) families (leucine, valine, alanine, creatine, glycine, 5-methylhistidine, methionine), four carbohydrate families (glucose, lactose, sucrose, galactarate), two carboxylic acid families (formate, lactate), one methoxyphenol family (thymol), two alcohol families (methanol, pantothenate), and seven others (succinylacetone, acetoacetate, Citrate, ascorbate, 4-pyridoxate, levulinate, 3-hydroxybutyrate). A total of 23 metabolites showed correlations with immune indicators. Among them, nine metabolites exhibited a positive correlation with immune indicators that increased during high-temperature periods, while 14 metabolites showed a negative correlation. Methionine and thymol were correlated exclusively with IL-10 (p<0.05), with no significant correlation observed with other immune indicators. Similarly, leucine was correlated only with IL-17A (p<0.05), while lactate, succinylacetone, and 4-pyridoxate were associated solely with B cells (p<0.05). Sucrose and galactarate showed correlations only with CD4+ T cells (p< 0.05), whereas acetoacetate was correlated exclusively with CD4+ IL-17+ cells (p<0.05) (Figure 9).

## DISCUSSION

Conditions of high THI impose severe negative effects on livestock productivity by dissipating heat and causing stress, which exceeds the physiological ultimate [21]. Livestock exposed to HS may have a negative energy balance (NEB) caused by reduced feed intake and undergo energy redistribution processes, which change the metabolic response and elevate insulin and protein catabolism in the blood [22]. Therefore, HS exposure increases the risk of NEB and impairs postabsorptive energy metabolism, directly and negatively affecting livestock energetics [3,23]. Due to higher metabolic rates and lower body heat production, dairy cows have a higher sensitivity to HS than beef cattle [24]. Therefore, the demand for innovative strategies to prevent economic losses and metabolic impairments in dairy cows is high. To this end, understanding the metabolic changes in dairy cows caused by HS is essential in order to develop effective strategies.

Acetate, propionate, and butyrate are the main volatile fatty acids (VFAs) produced during rumen microbial fermentation and are associated with feed efficiency, influencing ruminant growth and production. Several studies have shown a negative effect of HS on rumen VFA concentrations, which may be associated with abnormal changes in rumen metabolism from reduced feed intake [25], increased water intake, and altered rumen microbial population [26]. In dairy cows, acetate and propionate are important substrates for milk fat and lactose synthesis, respectively, and are strongly associated with gluconeogenesis [27]. In this study, acetate and propionate concentrations in rumen fluid were significantly higher in OTP than in HTP. In addition, glycolysis and gluconeogenesis were significantly different (p<0.0001). Glycolysis pathway is crucial for energy supply in all cells, it plays a particularly vital role in immune cells. Immune cells often rely on glycolysis even in the presence of oxygen. The metabolic switch is not only for meeting energy demands but also supports rapid cellular functions that are critical during immune responses. Moreover, the by-products of glycolysis, such as lactate, play regulatory roles in the immune environment. High levels of lactate can inhibit the proliferation and function of other immune cells, modulating the inflammatory response. In this study, the decrease in lactate levels during the HTP may be associated with the increased neutrophil counts. Heat-stressed animals occasionally have lower blood glucose concentrations, which affects glycolysis and gluconeogenesis [8]. During HS in dairy cows, downregulation of serum and plasma glucose concentrations have been observed with increased in the AAs concentration involved in gluconeogenesis [28]. HS in dairy cows has been associated with elevated levels of AAs involved in gluconeogenesis [28]. Similarly, steers subjected to HS have exhibited higher levels of glucogenic AAs, including alanine, glutamine, methionine, phenylalanine, proline, tyrosine, and valine [29,30]. Okoruwa et al reported increased concentrations of total protein and albumin in goats under HS, which could be attributed to dehydration resulting from an increased respiration rate [29]. Altered serum metabolic pathways, including glycolysis and AA metabolism, have been observed in steers [30]. These results suggest that HS exposure increases AA metabolism to compensate for reduced glucose concentration in dairy cows.

Alcohol dehydrogenase metabolizes isopropanol to acetone, acetol, methylglyoxal, propylene glycol, acetate, and formate, which are further converted to glucose and other intermediate metabolic products [31]. In this study, formate concentration was significantly higher in HTP than in OTP (p<0.0001 and VIP score: 2.61). Formate is an essential endogenous one-carbon metabolite in animals that participates in a vital one-carbon pool of intermediary metabolism and is a valuable biomarker for impaired one-carbon metabolism [32]. During nucleic acid biosynthesis, formate provides a carbon source for N5- and N10-methylenetetrahydrofolate in the blood. In addition, formate can be used to treat inflammatory conditions in dairy cows involving purine nucleotide biosynthesis, such as mastitis [33]. Yue et al reported a high formate concentration in HS period, suggesting that impaired one-carbon metabolism and possible signs of metabolic acidosis diseases [34]. Therefore, dairy cows exposed to HS are expected to show changes in formate concentration in the serum, as exposure to HS may lead to an increase in metabolic diseases, such as acidosis. In addition, isopropanol and acetone are used to diagnose human diseases associated with intoxication [35]. Andersson identified isopropanol (C-3 alcohol) in the blood and various biofluids of cows experiencing ketosis, along with the presence of hyperketonemia [36]. In this study, isopropanol concentration was significantly higher in HTP than that in OTP (p<0.01; VIP score: 1.89). Moreover, HTP exhibited a high acetone concentration (p<0.01; VIP score: 2.01) which is a metabolite of ketone bodies. Ketosis can have detrimental consequences, such as decreased milk production, impaired reproductive performance, elevated susceptibility to lameness, mastitis, metritis, retained placenta, and an increased likelihood of culling [37,38]. During summer, ruminants are prone to ketosis because of heightened maintenance needs, challenges in thermoregulation, and reduced feed consumption [39]. Hence, isopropanol and acetone presence in the blood of dairy cows could serve as indirect indicators of HS.

Numerous studies have identified the metabolic biomarkers of milk composition for HS diagnosis. Dairy cows utilize glucose as an energy source [40], in addition to a building block for lactose synthesis in milk. Within the mammary glands, a single glucose molecule is transformed into galactose, which subsequently combines with another glucose molecule to facilitate lactose. Lactose is a major sugar in milk that is affected by the reshuffling of whole body energy metabolism during HS, which contributes to milk production loss [8]. In this study, lactose concentration was significantly higher in OTP than that in HTP (p<0.01, VIP score: 1.60). Glycine could protect diabetic β-cells against damage caused by oxidative stress by increasing glycine transporter-1-mediated synthesis of glutathione and by reducing glycine receptor-mediated reactive oxygen species production [41]. Klein et al [42] reported that early lactation implies increased glycine metabolism, which results in higher milk production in dairy cows. In addition, glycine can provide energy for the metabolism of mammary gland cells and act as a precursor for protein synthesis and enzyme synthesis. In addition, glycine is involved in glyoxylate and dicarboxylate metabolism, where cis-aconitate and glycine are converted into each other. Glucitol (less commonly known as sorbitol) is an intermediate in the metabolism of fructose, mannose, and galactose and plays a role in transporting sugar as a glucose and fructose (associated milk lactose concentration) precursor in milk. Heat-stressed dairy cows have reduced DMI, milk yield, milk protein, and milk, urea, and nitrogen contents but increased milk somatic cell counts [43]. Bobbo et al [44] reported that lactate, phenylalanine, choline, acetate, O-acetylcarnitine, 2-oxoglutarate, and valine in milk samples containing high levels of somatic cells. In this study, glucitol concentration was significantly higher in OTP than that in HTP (p<0.001 and VIP score: 1.80). In addition, fructose and mannose metabolism were significantly different (p<0.001) in this study. Moreover, choline concentration was significantly higher in HTP than that in OTP (p<0.05).

In this study, ethanol is one of the major metabolites altered in the rumen under HS. HS cows had higher ethanol concentration in that HS-free cow. Ethanol is a physiological factor that has the potential to affect the gastrointestinal barrier function and promote the translocation of endotoxins into the bloodstream, triggering an inflammatory response. Previous studies have proposed that ethanol produced in the rumen could play a role in facilitating endotoxin translocation across the walls of the rumen and colon, thereby contributing to the development of endotoxin-related disorders in dairy cows [45]. These results may suggest that metabolic changes by HS may affect immunological status in dairy cows.

HS induces various physiological responses, which result in altered immunity such as immune cell composition which may increase susceptibility to both infectious and noninfectious diseases. Previous correlation studies have found that livestock experiencing HS had a higher incidence of infectious and metabolic diseases [3,46]. Various stress-related molecules affect immunological response, which results in increased susceptibility of animals. Therefore, we isolated PBMCs from blood and conducted flow cytometric analysis for assessing immune cells [47]. Monocytes are important innate immune cells in the blood of animals. PBMCs contain B and T cells, which are essential for cellular and humoral immune responses. In this study, the proportion of CD21+ MHCII + B cells was significantly affected by HS conditions. B cells produce antibodies and play an important role in preventing the spread of infection in the body. In some cases, excessive B cell response induces inflammatory diseases in animals. Other studies have reported that animals exposed to HS experienced problems with B lymphocyte differentiation and proliferation [48,49], and HS decreased immunoglobulin levels [50]. The B cell population increased in Jersey cows under HS conditions in this study. This result is consistent with a previous study showing that the population of B cells tends to increase during HTP [51]. In this study, we did not examine the functional response of B cells, such as antibody production, so we could not determine the outcome of increased B cell numbers in blood. Serum antibody levels need to be determined in future study.

Among the lymphocytes, T cells directly eliminate infected host cells, activate other immune cells, and regulate the immune response by producing cytokines. Changes in CD4+ and CD8+ T cell counts are potential biomarkers of HS in livestock. Several studies have reported that HS conditions increase the number of CD4+ T cells, which is consistent with our observations. As CD4+ T cells are influenced by HS, we further examined CD4+ T cell subsets such as Th1 and Th17 cells. The pro-inflammatory cytokine IL-17A induces inflammation by promoting the expression of cytokines, chemokines, and anti-microbial peptides. IL-17A is involved in allergies, autoimmune diseases, and can mediate protective innate immunity against pathogens. In this study, HS increased the number of Th17 cells. Also, qRT-PCR analysis showed elevated IL-17A gene expression during HTP. IL-17A was reported to enhance B cell survival and proliferation and differentiation into plasma cells [52]. In addition, previous reports suggest that cytokines produced by Th17 cells induce effective B-cell differentiation, [53]. Thus, these results suggested possibility that an increase in Th17 (IL-17A+CD4+) cells in Jersey cows exposed to HS may affect B cell survival and proliferation. In contrast of increased pro-inflammatory cytokine IL-17A, we observed anti-inflammatory cytokine IL-10 gene expression decreased during HTP. In general, dairy cows in HTP showed increased inflammatory response based on cytokine expression [3,46,53,54]. Inflammatory conditions may link to various disease development including metabolic disease in dairy cows during HTP.

Metabolites are a critical factor for not only host metabolism but also, immunity in animals. In fact, accumulating data suggests that various metabolites significantly influence immune cell function and differentiation. For example, various types of AAs, cholesterol, and fatty acids affect immune activities [55–57]. A lack of metabolites in the body affects almost all immune conditions, particularly nonspecific deficiencies and cell-mediated immunity. In particular, micronutrients such as iron and zinc are impaired by pathogen [58]. Iron and zinc levels rapidly decline during infection associated with inflammation. Therefore, we examined possible connection between metabolites and immune parameters, that changed by HS condition. We analyzed the correlation between the serum metabolite concentration and proportion of immune cells in the blood that are changed by environmental condition. The B cells, IL-17A expression in PBMCs, total CD4+ T cells, and Th17 cells elevated upon exposure to HTP displayed similar trends in the correlation plot, suggesting a strong association between them. The levels of various AA metabolites, such as valine, alanine, creatine, and glycine were reduced by HS and showed a strong correlation with immune parameters.

Glycine has anti-inflammatory, immunomodulatory, and protective effects. Furthermore, glycine is reported to reduce pro-inflammatory cytokine expression and increase anti-inflammatory cytokine expression through NF-kB inhibition [59]. In this study, the low blood concentration of glycine and increase Th17 response in HTP condition observed. It may suggest that low glycine levels may influence the reduced inhibitory effect of pro-inflammatory cytokine such as IL-17A. Moreover, the decrease in anti-inflammatory IL-10 gene expression was expected to be influenced by glycine during HTP. In our study, valine showed negative correlation to Th17 cells and positive correlation and IL-10. Inflammation triggers various responses leading to the release of pro-inflammatory cytokines. Inflammation-saturated nitric oxide (NO) causes cell death through oxidative stress, energy metabolism disruption, and DNA damage. Valine decreased NO production, thus reducing inflammation and regulating iNOS expression to control DNA damage, mutations, and oxidative stress. Decreased valine concentration in serum during the HTP may have affected the regulation of NO expression.

Alanine is an AA and is a source of energy for muscles and the central nervous system. Additionally, alanine is known to enhance the immune system, exerting significant influence on T cells [60]. Activated T cells are metabolically demanding, and various nutrients, such as glucose, leucine, and arginine are essential for T cell activation [61]. Alanine deficiency results in inhibited T cell metabolism and effector functions. Ron-Harel et al reported that alanine is required for efficient naïve T cell activation and memory T cell restimulation [61]. Alanine deficiency in blood during HTP may affect T cell activation and differentiation in cows, potentially leading to immune suppression. Although alanine can be synthesized from pyruvate, which is mainly derived from glucose, decreased blood glucose levels during HTP may lead to insufficient alanine production. Additionally, glucose is crucial for cell metabolism and therefore can have a significant impact on the immune cells of cows. Although, we do not know exact mechanism for alanine regulation of immune response, reduced alanine may affect immune cell activation.

Glucose is an essential nutrient for all cells and serves as the primary source of energy. Low glucose levels in the body can trigger changes such as platelet activation and leukocytosis. Low glucose levels lead to an increase in IL-1B in monocytes, and this result was amplified in the presence of lipopolysaccharide. Therefore, decreased glucose levels during HTP may induce a sensitive inflammatory response in heat-stressed Jersey [62]. Thymol has anti-inflammatory effects by inhibiting the release of inflammatory cytokines from macrophages and downregulating signaling pathways such as NF-κB and MAPK [63,64]. Additionally, thymol inhibits the reduction of myeloperoxidase in neutrophils and the release of elastase, thereby exerting anti-inflammatory effects [64,65]. In our study, thymol exhibits a positive correlation with IL-10, indicating that the anti-inflammatory effects of thymol may decrease during HTP.

Although, we identified several metabolites that show strong correlation to immune parameters, that affected by HS. It really notes that functional roles of the identified metabolites in immune response of dairy cow really provide useful information for development of nutritional strategies. It is may be possible to do in vitro immune cell culture experiments with identified metabolites in future study. However, we postulate that one metabolite play a key role in induction of pro-inflammatory phenotype of dairy cow during HTP. It assume that HS-sensitive metabolome works together to make abnormal immune response in dairy cow. *In vitro* studies to determine the immunological functions of individual metabolites need to be conducted in future research. This follow-up study will provide useful information about nutritional approach to manipulate immune response in heat-stressed cow.

In summary, high-temperature stress increased CD4+ T cells in Jersey cows, with a particular increase in the distribution of T cells expressing IL-17A. This phenomenon is associated with an increase in the number of B cells and may lead to excessive inflammatory responses during HTPs. T cell differentiation and an increase in B cells consume significant energy, resulting in the consumption of many metabolites, including glucose. Coupled with reduced feed intake during summer, this process can induce hypoglycemia and potentially affect health.

Overall, these results indicate that altered metabolic profile by HS condition may influence excessive pro-inflammatory cytokine production in Jersey cows, leading to an imbalance in immune regulation and potential over activity of pro-inflammatory cells.

Our study has several limitations. The sample size is relatively small, making it difficult to generalize the findings to all Jersey cows. Future studies with larger numbers of experimental animals and more sampling time points to validate findings are needed to enhance generalizability of findings. Moreover, long-term observations could help in better understanding the relationship between metabolites and immune cells. Based on these findings, it is possible to develop targeted nutritional interventions to alleviate the negative effects of HS on immune function. For instance, alanine, that HS-sensitive metabolite due to its potential known role in immune regulation, could be considered a potential HS-specific feed additive to mitigate immune imbalances induced by HS. Future studies on the microbiome should explore how HS-induced alterations in microbial communities contribute to metabolic changes and, in turn, impact immune indicators.

## CONCLUSION

In this study, we found that altered metabolism and immunological responses of Jersey cows under HS conditions by comparing metabolites and immune cell proportions and gene expression in the blood during optimum and high temperature conditions (OTP and HTP, respectively). Isopropanol, formate, threonine, glycine, glucitol, trehalose, and 3-phenylpropionate metabolites were significantly altered during HS, suggesting their potential as biomarkers for HS-induced metabolic changes in Jersey cows, and HS-induced immune responses were characterized by increased levels of IL-17A, IL-10, B cells, and CD4+ T cells. An integrated perspective of change in serum metabolite and immune cells in Jersey cows demonstrated a strong correlation between serum metabolites such as glycine, alanine, and glucose with the regulation of immune cell differentiation and function. Conclusively, these findings provide a basis for developing biomarker-based diagnostic tools for HS in dairy cows. Identifying metabolic alterations under HS enables the development of targeted nutritional interventions to mitigate its negative effects on metabolism and immunity.

## Figures and Tables

**Table 1:** Chemical composition of experimental diets

Chemical composition^1)^	Total mixed ration
Dry matter (%)	63.5
Moisture (%)	36.5
Nutrient composition (% of dry matter)	
Crude protein	16.2
Soluble protein	6.4
Acid detergent fiber	23.2
Neutral detergent fiber	42.4
Starch	14.5
Lignin	4.23
Total digestible nutrients	67.4
Minerals (% of dry matter)	
Ash	8.81
Calcium	1.08
Phosphorus	0.48
Magnesium	0.45
Potassium	1.59
Sodium	0.32
Iron (ppm)	551.0
Manganese (ppm)	119.0
Zinc (ppm)	81.0
Copper (ppm)	22.0
Energy (Mcal/kg)	
Net energy lactation	1.52
Net energy maintenance	1.65
Net energy gain	1.04
Metabolizable energy	2.56

1) Chemical analysis was performed by Cumberland Valley Analytical Services (Waynesboro, PA, USA).

## SUPPLEMENTARY MATERIAL

**Supplement 1**. Identified metabolites in the rumen fluid (A), serum (B), and milk (C) from Jersey cows under OTP and HTP conditions.

**Supplement 2**. Quantified metabolites in the rumen fluid (A), serum (B), and milk (C) from Jersey cows under OTP and HTP conditions.

**Supplement 3**. The numbers of unique and common identified (A) and quantified (B) metabolites from Jersey cows under OTP and HTP conditions.

**Supplement 4**. Differential enrichment of metabolites contents of Jersey cow’s rumen fluid between optimum temperature period and high temperature period conditions.

**Supplement 5**. Differential enrichment of metabolites contents of Jersey cow’s serum between optimum temperature period and high temperature period conditions.

**Supplement 6**. Differential enrichment of metabolites contents of Jersey cow’s milk between optimum temperature period and high temperature period conditions.

**Supplement 7**. Pathway analysis significantly different rumen fluid metabolites compared with optimum temperature period and high temperature period conditions.

**Supplement 8**. Pathway analysis significantly different serum metabolites compared with optimum temperature period and high temperature period conditions.

**Supplement 9**. Pathway analysis significantly different milk metabolites compared with optimum temperature period and high temperature period conditions.

**Supplement 10**. The The number of immune cells in the blood of Jersey cows during OTP and HTP conditions.
