# Supplementary material for: Integrated metabolome and immunity analysis of immune-physiological responses in dairy cows under heat stress condition
Source: Anim Biosci. 2025 May 12;38(10):2215–32. doi: 10.5713/ab.25.0038 (PMC12415360; doi:10.5713/ab.25.0038)
Supplement: Supplementary file 2 [file ab-25-0038-Supplementary-2.pdf]

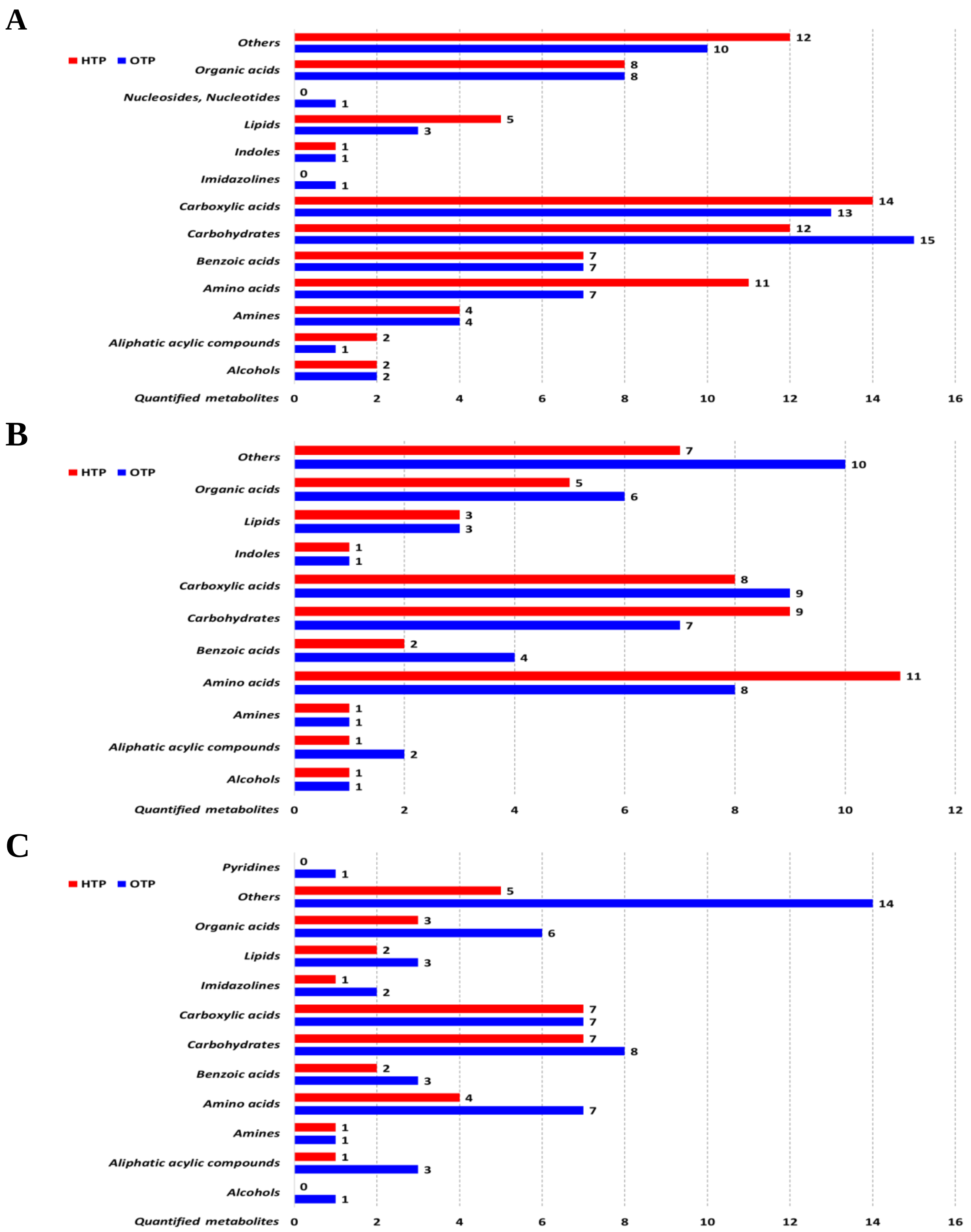

Supplement 2. Quantified metabolites in the rumen fluid **(A)**, serum **(B)**, and milk **(C)** from Jersey cows under OTP and HTP conditions.
