# Supplementary material for: Integrated metabolome and immunity analysis of immune-physiological responses in dairy cows under heat stress condition
Source: Anim Biosci. 2025 May 12;38(10):2215–32. doi: 10.5713/ab.25.0038 (PMC12415360; doi:10.5713/ab.25.0038)
Supplement: Supplementary file 3 [file ab-25-0038-Supplementary-3.pdf]

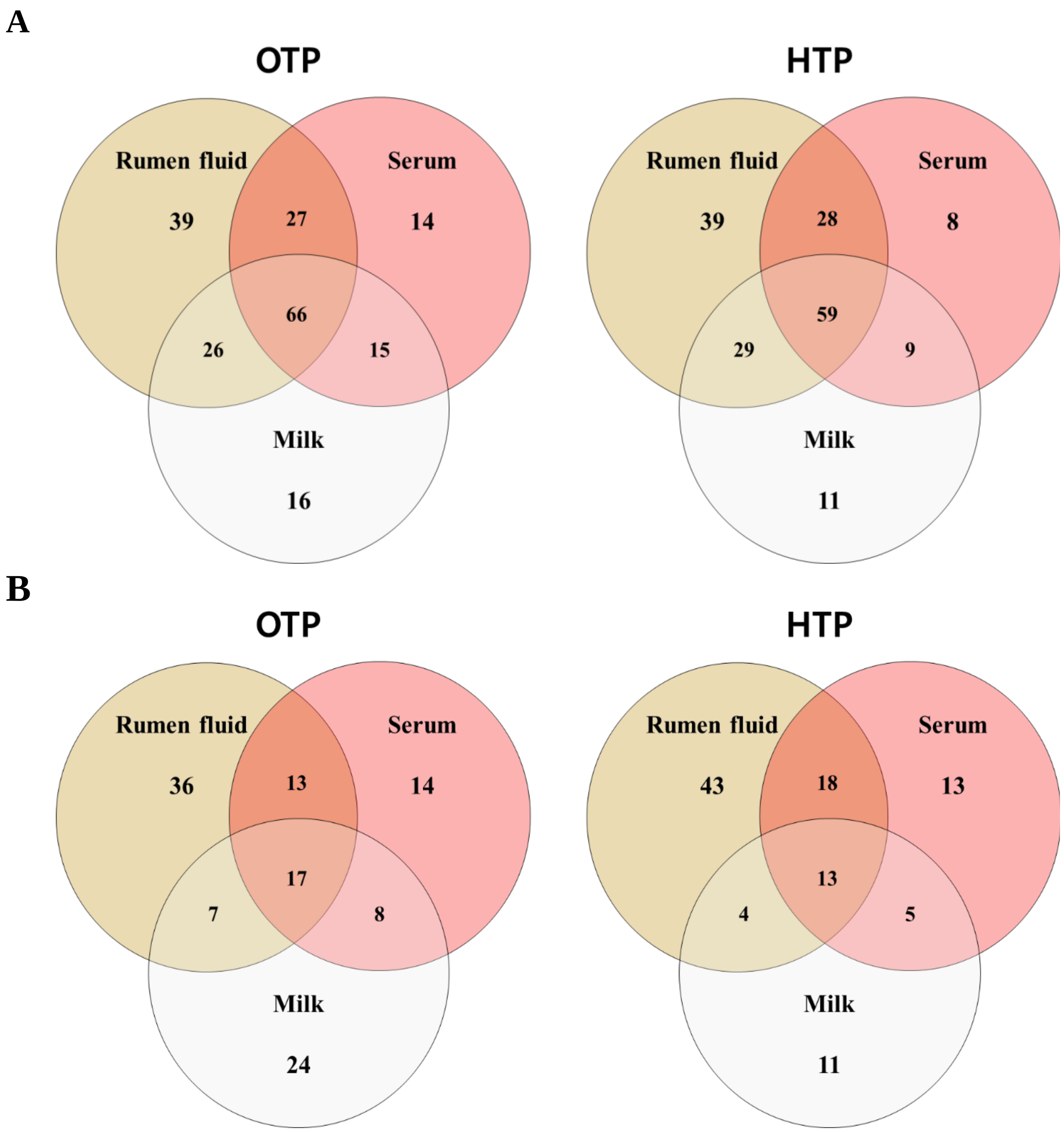

Supplement 3. The numbers of unique and common identified **(A)** and quantified **(B)** metabolites from Jersey cows under OTP and HTP conditions.
