# Supplementary material for: Integrated metabolome and immunity analysis of immune-physiological responses in dairy cows under heat stress condition
Source: Anim Biosci. 2025 May 12;38(10):2215–32. doi: 10.5713/ab.25.0038 (PMC12415360; doi:10.5713/ab.25.0038)
Supplement: Supplementary file 4 [file ab-25-0038-Supplementary-4.pdf]

Supplement 4. Differential enrichment of metabolites contents of Jersey cow's rumen fluid between optimum temperature period and high temperature period conditions

Metabolites	Class[1]	*P* value[2]	VIP[3]	FC[4]	OTP vs HTP
Trimethylamine	Amine	1.31×10^{-7}	2.11	-1.73	HTP
Ethanol	Alcohol	7.47×10^{-6}	1.98	-1.26	HTP
Nicotinate	OA	1.14×10^{-5}	1.96	0.96	OTP
N-methylhydantoin	Other	1.20×10^{-5}	1.96	-1.08	HTP
4-aminobutyrate	AA	1.61×10^{-5}	1.94	-1.04	HTP
Valine	AA	4.77×10^{-5}	1.89	-0.79	HTP
Uracil	ns & nt	9.01×10^{-5}	1.85	0.86	OTP
Imidazole	IMI	1.86×10^{-4}	1.80	1.36	OTP
Maltose	CHO	2.96×10^{-4}	1.76	-0.68	HTP
Acetate	OA	5.18×10^{-4}	1.72	0.37	OTP
Choline	Lipid	6.29×10^{-4}	1.70	-1.07	HTP
Hypoxanthine	ns & nt	7.20×10^{-4}	1.69	0.75	OTP
Melatonin	Other	7.21×10^{-4}	1.69	-0.54	HTP
Benzoate	OA	7.75×10^{-4}	1.68	0.93	OTP
Taurine	Amine	1.11×10^{-3}	1.65	0.58	OTP
Gentisate	BZA	1.40×10^{-3}	1.63	0.84	OTP
Trimethylamine *N*-oxide	A.comp	1.40×10^{-3}	1.63	-0.91	HTP
Propionate	OA	1.48×10^{-3}	1.62	0.32	OTP
3-phenylpropionate	Other	1.61×10^{-3}	1.61	0.50	OTP
Dimethyl sulfone	Other	2.06×10^{-3}	1.58	0.83	OTP
Erythritol	CHO	2.13×10^{-3}	1.58	1.05	OTP
1,3-dimethylurate	Other	2.38×10^{-3}	1.57	-0.53	HTP
2-phenylpropionate	COOH	2.47×10^{-3}	1.57	0.59	OTP
Glucose	CHO	2.50×10^{-3}	1.56	-0.55	HTP
2-oxocaproate	Other	2.55×10^{-3}	1.56	0.50	OTP
N-nitrosodimethylamine	OA	3.08×10^{-3}	1.54	-0.57	HTP
Threonate	CHO	3.34×10^{-3}	1.53	0.55	OTP
Methanol	Alcohol	3.36×10^{-3}	1.53	-0.63	HTP
4-hydroxyphenylacetate	BZA	3.72×10^{-3}	1.52	0.61	OTP
5-methoxysalicylate	BZA	3.92×10^{-3}	1.51	0.61	OTP
Butyrate	OA	4.26×10^{-3}	1.50	0.28	OTP
Kynurenate	Other	4.93×10^{-3}	1.48	0.60	OTP
Guanidoacetate	COOH	5.33×10^{-3}	1.47	-0.79	HTP
Isovalerate	OA	5.95×10^{-3}	1.46	0.39	OTP
Xanthurenate	AA	6.46×10^{-3}	1.45	0.67	OTP
N-isovaleroylglycine	AA	7.61×10^{-3}	1.42	0.81	OTP
β-alanine	AA	7.81×10^{-3}	1.42	-0.48	HTP
5-hydroxytryptophan	Indole	8.40×10^{-3}	1.41	0.46	OTP
Isopropanol	Alcohol	9.82×10^{-3}	1.39	0.36	OTP
Thymine	Pyridine	1.08×10^{-2}	1.37	0.57	OTP
Pantothenate	COOH	1.36×10^{-2}	1.34	-0.42	HTP
1,3-dihydroxyacetone	CHO	1.46×10^{-2}	1.33	0.65	OTP
Anserine	AA	1.47×10^{-2}	1.33	-0.43	HTP
p-cresol	BZA	1.50×10^{-2}	1.32	0.54	OTP
1,7-dimethylxanthine	Other	1.52×10^{-2}	1.32	-0.55	HTP
Ethylene glycol	Lipid	1.54×10^{-2}	1.32	-0.51	HTP

Gallate	BZA	1.58×10^{-2}	1.31	0.26	OTP
2-furoylglycine	AA	1.74×10^{-2}	1.30	0.26	OTP
N-phenylacetylphenylalanine	COOH	1.85×10^{-2}	1.29	0.41	OTP
2-hydroxy-3-methylvalerate	Lipid	1.86×10^{-2}	1.29	0.41	OTP
Valerate	OA	2.20×10^{-2}	1.26	0.21	OTP
Caprylate	Lipid	2.23×10^{-2}	1.26	0.49	OTP
Homogentisate	BZA	2.44×10^{-2}	1.24	0.70	OTP
Dimethylamine	Amine	2.79×10^{-2}	1.22	-0.67	HTP
Salicylurate	BZA	2.82×10^{-2}	1.22	0.53	OTP
Leucine	AA	2.90×10^{-2}	1.21	-0.39	HTP
4-guanidinobutanoate	COOH	3.31×10^{-2}	1.19	0.64	OTP
Alloisoleucine	COOH	3.42×10^{-2}	1.18	0.24	OTP
Isoleucine	AA	3.44×10^{-2}	1.18	-0.59	HTP
Phenylacetate	OA	4.75×10^{-2}	1.11	0.17	OTP

[1]Class abbreviations: AA, amino acid; A.comp, aliphatic acylic compound; BZA, benzoic acid; CHO, carbohydrate; COOH, carboxylic acid; IMI, imidazolinone; ns & nt, nucleoside & nucleotide; OA, organic acid

[2]Significant difference as determined by the Student's t-test model ($P < 0.05$)

[3]Variable importance in the projection (VIP) score was obtained from partial least squares-discriminant analysis model

[4]Fold change (FC) was calculated as binary logarithm of average concentration response ratio between optimum temperature period (OTP; n = 9) and high temperature period (HTP; n = 8) conditions, where the positive value means that the average concentration response of the metabolite in the former is larger than that in the latter and vice versa
