# Supplementary material for: Integrated metabolome and immunity analysis of immune-physiological responses in dairy cows under heat stress condition
Source: Anim Biosci. 2025 May 12;38(10):2215–32. doi: 10.5713/ab.25.0038 (PMC12415360; doi:10.5713/ab.25.0038)
Supplement: Supplementary file 5 [file ab-25-0038-Supplementary-5.pdf]

Supplement 5. Differential enrichment of metabolites contents of Jersey cow's serum between optimum temperature period and high temperature period conditions

Metabolites	Class[1]	*P* value[2]	VIP[3]	FC[4]	OTP vs HTP
Formate	OA	2.73×10^{-7}	2.61	0.35	OTP
Threonine	AA	2.73×10^{-4}	2.23	-0.79	HTP
Fucose	Other	3.37×10^{-4}	2.21	-1.28	HTP
Syringate	BZA	8.90×10^{-4}	2.11	-1.19	HTP
Acetone	Other	1.93×10^{-3}	2.01	-1.02	HTP
Sucrose	CHO	2.58×10^{-3}	1.97	-0.65	HTP
Galactarate	Other	4.51×10^{-3}	1.89	-0.56	HTP
Isopropanol	Alcohol	4.61×10^{-3}	1.89	-0.52	HTP
Glucose	CHO	4.64×10^{-3}	1.89	0.12	OTP
Methanol	Alcohol	5.06×10^{-3}	1.87	-1.10	HTP
Creatine	AA	7.10×10^{-3}	1.82	0.13	OTP
N-acetylglucosamine	CHO	8.97×10^{-3}	1.78	-0.39	HTP
Lactose	CHO	9.61×10^{-3}	1.76	0.36	OTP
Tyrosine	AA	1.10×10^{-2}	1.74	-0.45	HTP
Riboflavin	Other	1.22×10^{-2}	1.72	-0.40	HTP
Dimethylamine	Amine	1.87×10^{-2}	1.63	-0.80	HTP
Methylsuccinate	Lipid	2.10×10^{-2}	1.61	-0.43	HTP
Mannose	CHO	2.19×10^{-2}	1.60	0.55	OTP
Butanone	Other	2.41×10^{-2}	1.58	-0.45	HTP
Lactate	OA	3.11×10^{-2}	1.52	0.18	OTP
Alanine	AA	3.17×10^{-2}	1.52	0.09	OTP
Indole-3-acetate	Other	3.41×10^{-2}	1.50	0.69	OTP
Glycolate	Lipid	4.33×10^{-2}	1.44	-0.26	HTP
Glycine	AA	4.34×10^{-2}	1.44	0.33	OTP
Leucine	AA	4.36×10^{-2}	1.44	-0.18	HTP
Allantoin	IMI	4.45×10^{-2}	1.43	0.67	OTP
Indole-3-lactate	Other	4.73×10^{-2}	1.42	0.45	OTP
sn-glycero-3-phosphocholine	Other	4.92×10^{-2}	1.39	-0.57	HTP

[1]Class abbreviations: AA, amino acid; BZA, benzoic acid; CHO, carbohydrate; IMI, imidazolinone; ns & OA, organic acid

[2]Significant difference as determined by the Student's *t*-test model ($P < 0.05$)

[3]Variable importance in the projection (VIP) score was obtained from partial least squares-discriminant analysis model

[4]Fold change (FC) was calculated as binary logarithm of average concentration response ratio between optimum temperature period (OTP; $n = 9$) and high temperature period (HTP; $n = 8$) conditions, where the positive value means that the average concentration response of the metabolite in the former is larger than that in the latter and vice versa
