# Supplementary material for: Integrated metabolome and immunity analysis of immune-physiological responses in dairy cows under heat stress condition
Source: Anim Biosci. 2025 May 12;38(10):2215–32. doi: 10.5713/ab.25.0038 (PMC12415360; doi:10.5713/ab.25.0038)
Supplement: Supplementary file 6 [file ab-25-0038-Supplementary-6.pdf]

Supplement 6. Differential enrichment of metabolites contents of Jersey cow's milk between optimum temperature period and high temperature period conditions

Metabolites	Class[1]	*P* value[2]	VIP[3]	FC[4]	OTP vs HTP
Trehalose	CHO	1.91×10^{-6}	2.16	-1.30	HTP
1,3-dimethylurate	Other	1.28×10^{-5}	2.08	0.95	OTP
Glucuronate	CHO	6.44×10^{-5}	1.99	-1.22	HTP
4-methylhistidine	Other	1.03×10^{-4}	1.96	-1.11	HTP
2-hydroxyphenylacetate	Other	1.90×10^{-4}	1.92	-0.95	HTP
Pyruvate	CHO	1.94×10^{-4}	1.92	-1.26	HTP
2-ethylacrylate	Lipid	2.03×10^{-4}	1.92	-0.87	HTP
1,3-dihydroxyacetone	CHO	3.18×10^{-4}	1.88	-0.70	HTP
Galactonate	CHO	4.48×10^{-4}	1.85	1.54	OTP
3-hydroxymandelate	BZA	5.39×10^{-4}	1.84	-0.73	HTP
o-cresol	BZA	7.43×10^{-4}	1.81	-0.80	HTP
Glucitol	CHO	8.40×10^{-4}	1.80	0.55	OTP
Cellobiose	Other	1.14×10^{-3}	1.77	-0.57	HTP
Butanone	Other	1.19×10^{-3}	1.76	-0.69	HTP
Acetoin	Other	1.24×10^{-3}	1.76	-0.92	HTP
Urea	A.comp	1.28×10^{-3}	1.75	0.41	OTP
Guanidoacetate	COOH	1.41×10^{-3}	1.74	0.26	OTP
trans-aconitate	COOH	2.97×10^{-3}	1.66	-0.73	HTP
Glutamate	AA	3.15×10^{-3}	1.65	-0.63	HTP
Lactose	CHO	4.71×10^{-3}	1.60	0.25	OTP
Glucarate	CHO	5.50×10^{-3}	1.58	-0.54	HTP
Galactarate	Other	5.68×10^{-3}	1.58	0.85	OTP
Tartrate	BZA	6.58×10^{-3}	1.55	0.36	OTP
Theophylline	Other	6.91×10^{-3}	1.55	1.18	OTP
Alanine	AA	7.35×10^{-3}	1.54	-0.42	HTP
Glycine	AA	9.31×10^{-3}	1.50	0.20	OTP
Ethylene glycol	Lipid	9.32×10^{-3}	1.50	0.84	OTP
dTTP	ns & nt	1.54×10^{-2}	1.42	-0.55	HTP
cis-aconitate	COOH	2.76×10^{-2}	1.32	0.52	OTP
dCTP	ns & nt	2.88×10^{-2}	1.31	-0.25	HTP
Pyridoxine	Other	3.04×10^{-2}	1.30	-0.44	HTP
Choline	Lipid	3.94×10^{-2}	1.24	-0.45	HTP
3-hydroxyisovalerate	COOH	4.21×10^{-2}	1.23	-0.61	HTP
3-hydroxy-3-methylglutarate	Lipid	4.22×10^{-2}	1.23	-0.56	HTP

[1]Class abbreviations: AA, amino acid; A.comp, aliphatic acylic compound; BZA, benzoic acid; CHO, carbohydrate; COOH, carboxylic acid; ns & nt, nucleoside & nucleotide
[2]Significant difference as determined by the Student's *t*-test model ($P < 0.05$)
[3]Variable importance in the projection (VIP) score was obtained from partial least squares-discriminant analysis model
[4]Fold change (FC) was calculated as binary logarithm of average concentration response ratio between optimum temperature period (OTP; $n = 9$) and high temperature period (HTP; $n = 8$) conditions, where the positive value means that the average concentration response of the metabolite in the former is larger than that in the latter and vice versa
