# Supplementary material for: Integrated metabolome and immunity analysis of immune-physiological responses in dairy cows under heat stress condition
Source: Anim Biosci. 2025 May 12;38(10):2215–32. doi: 10.5713/ab.25.0038 (PMC12415360; doi:10.5713/ab.25.0038)
Supplement: Supplementary file 7 [file ab-25-0038-Supplementary-7.pdf]

Supplement 7. Pathway analysis significantly different rumen fluid metabolites compared with optimum temperature period and high temperature period conditions

Metabolic pathway	Hit/Total compounds[1]	Hit metabolites	*P* value	-log(*P*)	Impact[3]
Glycolysis / gluconeogenesis	3/26	Acetate, ethanol, glucose	9.32 × 10-8	7.03	0.03
beta-alanine metabolism	2/21	β-alanine, uracil	1.08 × 10-7	6.97	0.40
Pantothenate and CoA biosynthesis	4/19	Alanine, pantothenate, uracil, valine	7.33 × 10-7	6.14	0.03
Nicotinate and nicotinamide metabolism	1/13	Nicotinate	3.85 × 10-6	5.41	0.00
Arginine and proline metabolism	1/38	Guanidinoacetate	1.93 × 10-5	4.71	0.05
Pyrimidine metabolism	3/38	β-alanine, thymine, uracil	3.57 × 10-5	4.45	0.09
Glycine, serine and threonine metabolism	2/34	Choline, guanidinoacetate	3.77 × 10-5	4.42	0.02
Tryptophan metabolism	2/41	5-hydroxytryptophan, melatonin	1.10 × 10-4	3.96	0.16
Valine, leucine and isoleucine degradation	3/40	Isoleucine, leucine, valine	1.43 × 10-4	3.85	0.00
Valine, leucine and isoleucine biosynthesis	3/8	Isoleucine, leucine, valine	1.43 × 10-4	3.85	0.00
Aminoacyl-tRNA biosynthesis	3/48	Isoleucine, leucine, valine	1.43 × 10-4	3.85	0.00
Propanoate metabolism	1/23	β-alanine	5.55 × 10-4	3.26	0.00
Glycerophospholipid metabolism	1/36	Choline	6.16 × 10-4	3.21	0.03
Tyrosine metabolism	2/42	4-hydroxyphenylacetate, homogentisate	3.17 × 10-3	2.50	0.06
Purine metabolism	1/66	Hypoxanthine	3.64 × 10-3	2.44	0.02
Pyruvate metabolism	1/22	Acetate	1.17 × 10^{-2}	1.93	0.06
Glyoxylate and dicarboxylate metabolism	1/32	Acetate	1.17 × 10^{-2}	1.93	0.00
Caffeine metabolism	1/12	1,7-dimethylxanthine	1.49 × 10^{-2}	1.83	0.69
Taurine and hypotaurine metabolism	1/8	Taurine	1.86 × 10^{-2}	1.73	0.43
Primary bile acid biosynthesis	1/46	Taurine	1.86 × 10^{-2}	1.73	0.02

[1]Hit, the actually matched number from the user uploaded data; Total compounds, the total number of compounds in the pathway

[2]Impact, the pathway impact value calculated from pathway topology analysis
