# Supplementary material for: Integrated metabolome and immunity analysis of immune-physiological responses in dairy cows under heat stress condition
Source: Anim Biosci. 2025 May 12;38(10):2215–32. doi: 10.5713/ab.25.0038 (PMC12415360; doi:10.5713/ab.25.0038)
Supplement: Supplementary file 8 [file ab-25-0038-Supplementary-8.pdf]

Supplement 8. Pathway analysis significantly different serum metabolites compared with optimum temperature period and high temperature period conditions

Metabolic pathway	Hit/Total compounds[1]	Hit metabolites	*P* value	-log(*P*)	Impact[3]
Aminoacyl-tRNA biosynthesis	5/48	Alanine, glycine, leucine, threonine, tyrosine	5.80×10^{-6}	5.24	0.00
Alanine, aspartate and glutamate metabolism	1/28	Alanine	9.88×10^{-6}	5.01	0.00
Selenocompound metabolism	1/20	Alanine	9.88×10^{-6}	5.01	0.00
Glyoxylate and dicarboxylate metabolism	3/32	Formate, glycine, glycolate	1.37×10^{-5}	4.86	0.19
Glycine, serine and threonine metabolism	3/34	Creatine, glycine, threonine	1.39×10^{-5}	4.86	0.30
Arginine and proline metabolism	1/38	Creatine	2.06×10^{-5}	4.69	0.01
Glycolysis / gluconeogenesis	2/26	Glucose, lactate	3.58×10^{-5}	4.45	0.00
Pyruvate metabolism	1/22	Lactate	1.67×10^{-4}	3.78	0.00
Tryptophan metabolism	1/41	Indole-3-acetate	9.65×10^{-4}	3.02	0.00
Glutathione metabolism	1/28	Glycine	2.99×10^{-3}	2.53	0.09
Primary bile acid biosynthesis	1/46	Glycine	2.99×10^{-3}	2.53	0.02
Porphyrin and chlorophyll metabolism	1/30	Glycine	2.99×10^{-3}	2.53	0.00

[1]Hit, the actually matched number from the user uploaded data; Total compounds, the total number of compounds in the pathway

[2]Impact, the pathway impact value calculated from pathway topology analysis
