# Supplementary material for: Integrated metabolome and immunity analysis of immune-physiological responses in dairy cows under heat stress condition
Source: Anim Biosci. 2025 May 12;38(10):2215–32. doi: 10.5713/ab.25.0038 (PMC12415360; doi:10.5713/ab.25.0038)
Supplement: Supplementary file 9 [file ab-25-0038-Supplementary-9.pdf]

Supplement 9. Pathway analysis significantly different milk metabolites compared with optimum temperature period and high temperature period conditions

Metabolic pathway	Hit/Total compounds[1]	Hit metabolites	*P* value	-log(*P*)	Impact[3]
Glycolysis / gluconeogenesis	1/26	Pyruvate	2.69×10^{-5}	4.57	0.19
Arginine biosynthesis	2/14	Glutamate, urea	4.31×10^{-5}	4.37	0.12
Glyoxylate and dicarboxylate metabolism	4/32	cis-aconitate, glutamate, glycine, pyruvate	5.39×10^{-5}	4.27	0.13
Arginine and proline metabolism	3/38	Glutamate, guanidinoacetate, pyruvate	6.10×10^{-5}	4.21	0.11
Glycine, serine and threonine metabolism	4/34	Choline, guanidinoacetate, glycine, pyruvate	1.40×10^{-4}	3.85	0.30
Citrate cycle (TCA cycle)	2/20	cis-aconitate, pyruvate	2.17×10^{-4}	3.66	0.10
Fructose and mannose metabolism	1/20	Glucitol	8.02×10^{-4}	3.10	0.07
Glutathione metabolism	2/28	Glutamate, glycine	1.40×10^{-3}	2.86	0.11
Pentose and glucuronate interconversions	1/18	Glucuronate	2.14×10^{-3}	2.67	0.13
Alanine, aspartate and glutamate metabolism	3/28	Alanine, glutamate, pyruvate	2.16×10^{-3}	2.67	0.20
Pyruvate metabolism	1/22	Pyruvate	2.96×10^{-3}	2.53	0.21
Ascorbate and aldarate metabolism	2/10	Glucarate, glucuronate	3.08×10^{-3}	2.51	0.25
Primary bile acid biosynthesis	1/46	Glycine	1.95×10^{-2}	1.71	0.02
D-glutamine and D-glutamate metabolism	1/5	Glutamate	2.66×10^{-2}	1.58	1.00

[1]Hit, the actually matched number from the user uploaded data; Total compounds, the total number of compounds in the pathway

[2]Impact, the pathway impact value calculated from pathway topology analysis
