# Supplementary material for: Integrated metabolome and immunity analysis of immune-physiological responses in dairy cows under heat stress condition
Source: Anim Biosci. 2025 May 12;38(10):2215–32. doi: 10.5713/ab.25.0038 (PMC12415360; doi:10.5713/ab.25.0038)
Supplement: Supplementary file 10 [file ab-25-0038-Supplementary-10.pdf]

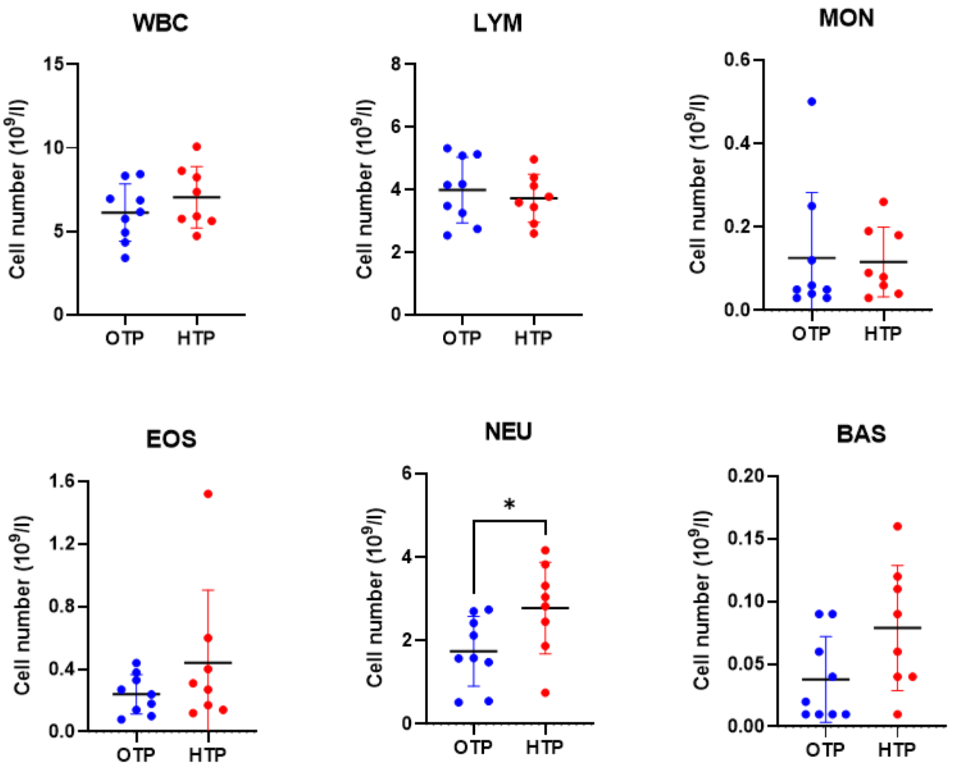

Supplement 10. The The number of immune cells in the blood of Jersey cows during OTP and HTP conditions. Whole blood samples were subjected to CBC analysis using the VetSacn HM5. Data are represented as means ± standard deviation (SD); n =8–9 animals/ group. WBC: white blood cell, LYM: lymphocyte, MON: monocyte, EOS: eosinophil, NEU: neutrophil, BAS: basophil. Values were statistically analyzed by Welch’s t test. * $p < 0.05$.
